# Plasma Biomarkers of Angiogenesis Related to Small Vessel Brain Disease in SPRINT

**DOI:** 10.1093/geroni/igab046.2517

**Published:** 2021-12-17

**Authors:** Nicholas Pajewski, Fanny Elahi, Joachim Ix, Ilya Nasrallah, Jason Hinman, Lenore Launer, Jeff Williamson, Donna Wilcock

**Affiliations:** 1 Wake Forest School of Medicine, Winston-Salem, North Carolina, United States; 2 University of California San Francisco, San Francisco, California, United States; 3 University of California San Diego, La Jolla, California, United States; 4 University of Pennslyvania, Philadelphia, Pennsylvania, United States; 5 David Geffen School of Medicine, University of California Los Angeles, Los Angeles, California, United States; 6 National Institute on Aging, Bethesda, Maryland, United States; 7 University of Kentucky, Lexington, Kentucky, United States

## Abstract

Meta-analyses incorporating the Systolic Blood Pressure Intervention Trial (SPRINT) have shown a reduced incidence of dementia with blood pressure lowering. However, mechanistic explanations for this effect are lacking, apart from slowed progression of cerebral white matter lesions (WML). Here we examine possible biomarkers of angiogenesis related to small vessel brain disease including bFGF, FLT1, PLGF, TIE-2, VEGF, VEGF-C, and VEGF-D. The biomarkers were assayed in plasma at baseline and during follow-up (median follow-up = 3.8 years) in a subgroup of participants 60 to 89 years old from SPRINT (N=517). We modeled changes in each biomarker using robust linear mixed models accounting for treatment group, time since randomization, and kidney function. Participants were 69.8 ± 7.1 (standard deviation) years of age, 42.1% female, with a mean systolic blood pressure (SBP) of 138.2 ± 17.0 mm Hg. At baseline, none of the biomarkers were associated with WML lesion volume or total brain volumes adjusting for age (all p>0.05), while FLT1, PLGF, and TIE-2 were negatively associated with frontal gray matter cerebral blood flow (partial correlations of -0.11, -0.10, and -0.12 respectively, all p<0.05). For both intensive (target SBP<120 mm Hg) and standard (target SBP<140 m Hg) blood pressure control, mean levels for the majority of biomarkers increased during follow-up, with the exceptions of TIE-2 (decreased over follow-up) and VEGF-D (no change). We did not observe significant between-group differences for the change in these plasma biomarkers of angiogenesis comparing intensive to standard blood pressure treatment.

